# Impact of surface chemistry of upconversion nanoparticles on time-dependent cytotoxicity in non-cancerous epithelial cells

**DOI:** 10.1038/s41598-024-83406-3

**Published:** 2024-12-23

**Authors:** Susanne Märkl, Frédéric Przybilla, Reinhard Rachel, Thomas Hirsch, Max Keller, Ralph Witzgall, Yves Mély, Joachim Wegener

**Affiliations:** 1 Institute for Analytical Chemistry, Chemo- and Biosensors, University of Regensburg, 93053 Regensburg, Germany; 2 Laboratory of Bioimaging and Pathologies, UMR 7021 CNRS, University of Strasbourg, Strasbourg, 67000 France; 3 Centre for EM, University of Regensburg, 93053 Regensburg, Germany; 4 Institute for Pharmacy, University of Regensburg, 93053 Regensburg, Germany; 5 Institute for Anatomy, University of Regensburg, 93053 Regensburg, Germany; 6 Fraunhofer Institute for Electronic Microsystems and Solid State Technologies EMFT, 93053 Regensburg, Germany

**Keywords:** Nanoscience and technology, Nanoscale materials, Nanotoxicology, Cytological techniques, Microscopy, Sensors and probes

## Abstract

The application of upconversion nanoparticles (UCNPs) for cell and tissue analysis requires a comprehensive understanding of their interactions with biological entities to prevent toxicity or harmful effects. Whereas most studies focus on cancer cells, this work addresses non-cancerous cells with their regular in vitro physiology. Since it is generally accepted that surface chemistry largely determines biocompatibility in general and uptake of nanomaterials in particular, two bilayer surface coatings with different surface shielding properties have been studied: (i) a phospholipid bilayer membrane (PLM) and (ii) an amphiphilic polymer (AP). Both surface modifications are applied to (12–33) nm core-shell UCNPs NaYF_4_(Yb, Er)@NaYF_4_, ensuring colloidal stability in biological media. The impact of UCNPs@AP and UCNPs@PLM on non-cancerous epithelial-like kidney cells in vitro was found to differ significantly. UCNPs@PLM did not exhibit any measurable effect on cell physiology, even with prolonged exposure. In contrast, UCNPs@AP caused changes in cell morphology and induced cell-death after approximately 30 h. These variations in toxicity are attributed to the distinct chemical stability of these particles, which likely influences their intracellular disintegration.

**Supplementary Information:**

The online version contains supplementary material available at 10.1038/s41598-024-83406-3.

## Introduction

Upconversion nanoparticles (UCNPs) have become a promising class of luminescent labels or probes to be used in the life sciences^1–4^. The lanthanide-doped nanocrystals show the outstanding ability to convert near-infrared (NIR) light into higher energy light in the visible (VIS) range of the spectrum^5^. The excitation with NIR light avoids autofluorescence of biomolecules and provides a better tissue penetration^6^. However, in a biological environment using biocompatible concentrations, particle brightness is limited as the luminescence efficiency of the UCNPs suffers from water quenching and particle disintegration comes into play^7–9^. We could show in a previous study that UCNPs modified with a phospholipid coating have the potential to solve these problems as they provide protection from water and as a consequence improved chemical stability^10^. Nevertheless, the particle dissolution inside living cells may lead to unknown interactions and trigger toxic responses. The released ions of metallic and metalorganic nanoparticles have been reported as primary source of nanotoxicity^11^ and need to be considered also for NaYF_4_-based UCNPs^12^. To date, most studies assessing the cytotoxicity of UCNPs report low toxicity. However, these findings are predominantly derived from end-point assays conducted within a limited concentration range and over short exposure times, typically focusing on a few cell types, mainly cancer cells^13–15^. Relying solely on end-point readouts to study cell fate upon UCNP exposure can easily produce false-negative results and oversimplifies the analysis. This approach fails to account for potential biotransformation of the particles and the biological impact of their disintegration products^14,15^. Therefore, a comprehensive investigation of cytotoxicity is essential, utilizing a combination of techniques that provide complementary information to capture a complete and accurate picture of UCNP-cell interactions over extended periods. The dissolution of NaYF_4_-based UCNPs in living cells has already been demonstrated to induce the formation of rare earth phosphates in lysosomes, and inflammatory responses^16^. This study has motivated researchers to improve the design of the UCNPs’ surface by hydrophobic bilayers, inorganic shells, or strongly complexing ligands for a better control of the particle/liquid interface and a reduced particle disintegration^17–24^. Nevertheless, most of these studies on UCNPs dissolution have been only performed under steady-state and cell-free conditions and need to be correlated to nanoparticle toxicity in vitro. When going from in vitro to in vivo applications, detailed studies on UCNPs cytotoxicity with long term exposures are essential. In this context, a suitable non-invasive technique for more detailed in vitro studies is electrical cell-substrate impedance sensing (ECIS)^25^. In such an assay, the cells are adherently grown on gold-film electrodes. The cell bodies block current flow as long as they are spread on the electrode surface and their membranes are intact. Once the cells shrink (apoptosis) or their membranes become permeable (necrosis), the electrical impedance drops, indicating cytotoxicity^25,26^. In parallel to such wholistic assays, a special focus should be directed to the potential interactions of the nanoparticles or their disintegration products with cellular organelles and molecular constituents. Importantly, studies should not only consider tumor cells but normal cells as well to avoid a biased perspective on the interactions of particles with tumor cells and their peculiar behaviors with respect to particle clearance and biodistribution^27^. Especially cell lines, derived from tissues forming the interface of the body to the outside world are of interest such as epithelial, endothelial, or phagocytic cells.

This work reveals the time-dependent impact of different surface coatings on nanoparticle uptake, stability, and long-time cytotoxicity in contact to non-cancerous epithelial-like cells. The surface coatings provide an individually efficient shielding from the aqueous environment. Upconversion nanoparticles of the type NaYF_4_(Yb, Er)@NaYF_4_have been selected as model systems as they show only very low dissolution tendency but a high optical sensitivity for changes that occur on the particle surface. These particles were coated with the bilayer forming and intrinsically non-toxic amphiphilic polymer (AP) or a phospholipid membrane (PLM) that mimics biological membranes as both have shown great potential in previous studies to provide protection from water^10^. This organic coating provides colloidal stability, reduces luminescence quenching, increases brightness and protects from chemical dissolution.

## Results and discussion

### Particle preparation and characterization

**Fig. 1:**
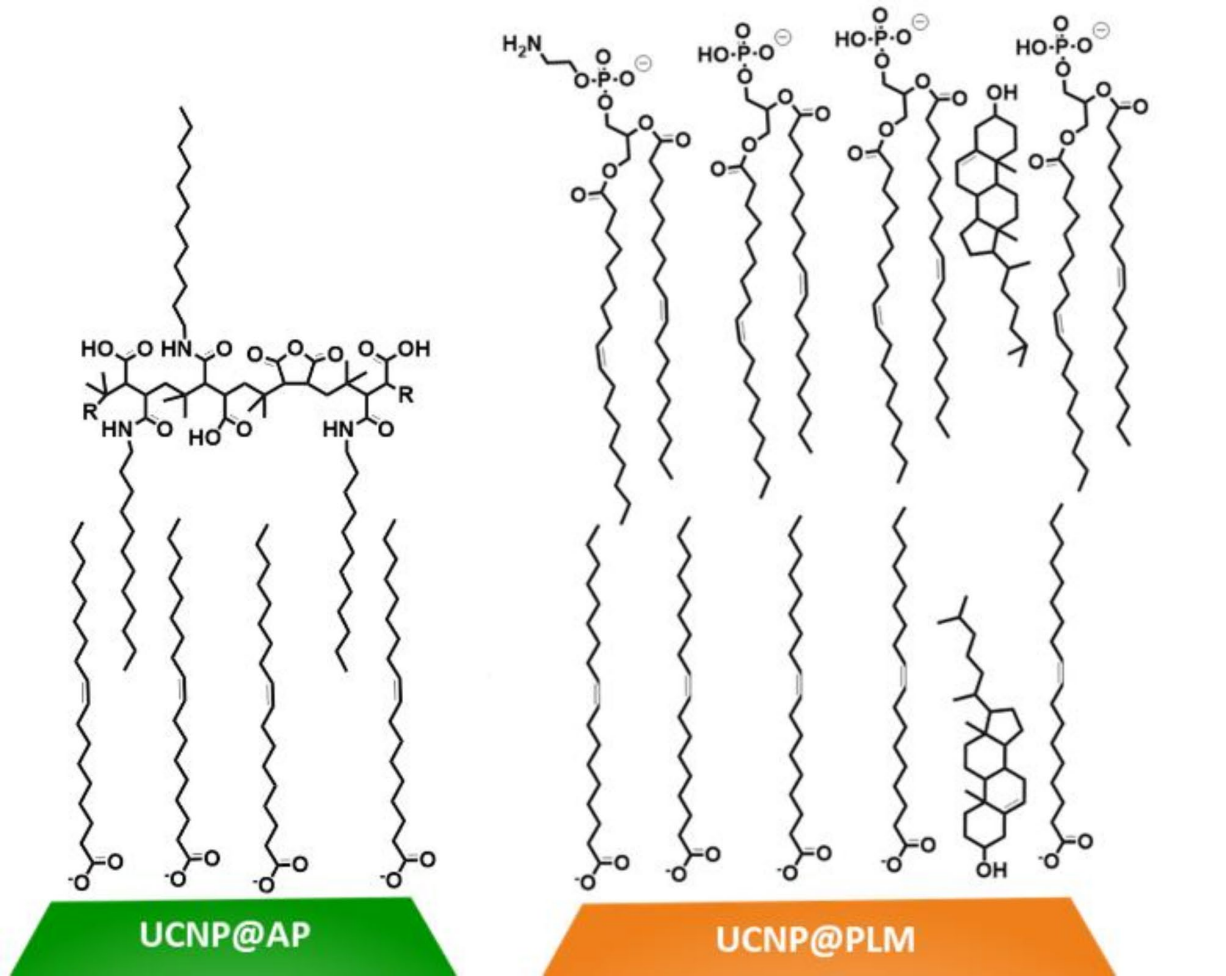
UCNPs were coated either with an amphiphilic polymer bilayer (UCNP@AP, green), most probably stabilized by intercalation, or with the phospholipid membrane coating (UCNP@PLM, orange). The PLM is formed by oleate, cholesterol, DOPA, and DOPE and, thereby, is stabilized by inter- and intramolecular Van der Waals interactions as well as electrostatic attraction of the head groups.

Particles consist of a NaYF_4_ core doped with 20% Yb^3+^ and 2% Er^3+^ (NaYF_4_(Yb, Er)). These particles cores are protected by a (2–3) nm optically inactive shell (NaYF_4_). After synthesis, the particles are stabilized by an oleate (OA) ligand and are denoted as UCNPs@OA. Hexagonal UCNPs@OA of two different diameters (12 ± 1) nm and (33 ± 1) nm have been used in this study (Figure S1 A, B). Both particles were modified with bilayer coatings consisting either of an amphiphilic polymer (AP; poly(isobutyl-maleic anhydride) with 75% dodecylamine side chains or a phospholipid membrane (PLM) made of 1,2-dioleoyl-sn-glycero-3-phosphate (DOPA, 64%), 1,2-dioleoyl-sn-glycero-3-phosphoethanolamine (DOPE, 7%) and cholesterol (29%). The negatively charged UCNPs@AP and UCNPs@PLM (Fig. 1) were prepared according to protocols reported previously^10,28,29^. All particles were proven to be monodisperse by TEM analysis, to be well dispersed and colloidally stable in H_2_O and cell culture media as verified by DLS, and to have a homogenous luminescence intensity distribution over a typical particle population under NIR excitation. The detailed results of the particle characterization are summarized in the supporting information, including TEM, DLS and zeta potential data, ensemble luminescence spectra, and single particle luminescence intensity distribution (Figure S1). An intrinsic toxicity of the PLM coating is excluded since the PLM bilayer consists of endogenous components of the mammalian cell membrane. The toxicity of the amphiphilic polymer (AP) was examined for NRK cells. AP forms aggregates with a hydrodynamic diameter of ~ 10 nm in water and of ~ 30 nm in fetal calf serum (FCS) containing buffers. NRK cells were exposed to the AP nanostructures in a dilution that was similar to that of the coated UCNPS (15 mM monomer concentration) and the cell response was monitored by ECIS (Figure S2). The impedance of the NRK cells does not decrease for at least 72 h upon exposure to AP agglomerates, indicating that cells remain unaffected by the presence of AP and show no sign of toxicity in this concentration range. Throughout this study, UCNPs of either 12–33 nm diameter were used. The larger UCNPs were used for optical studies to take advantage of their improved brightness and signal to noise ratio. Surface coatings were only compared for particles of the same size.

### Toxicity of UCNPs@AP and UCNPs@PLM

**Fig. 2:**
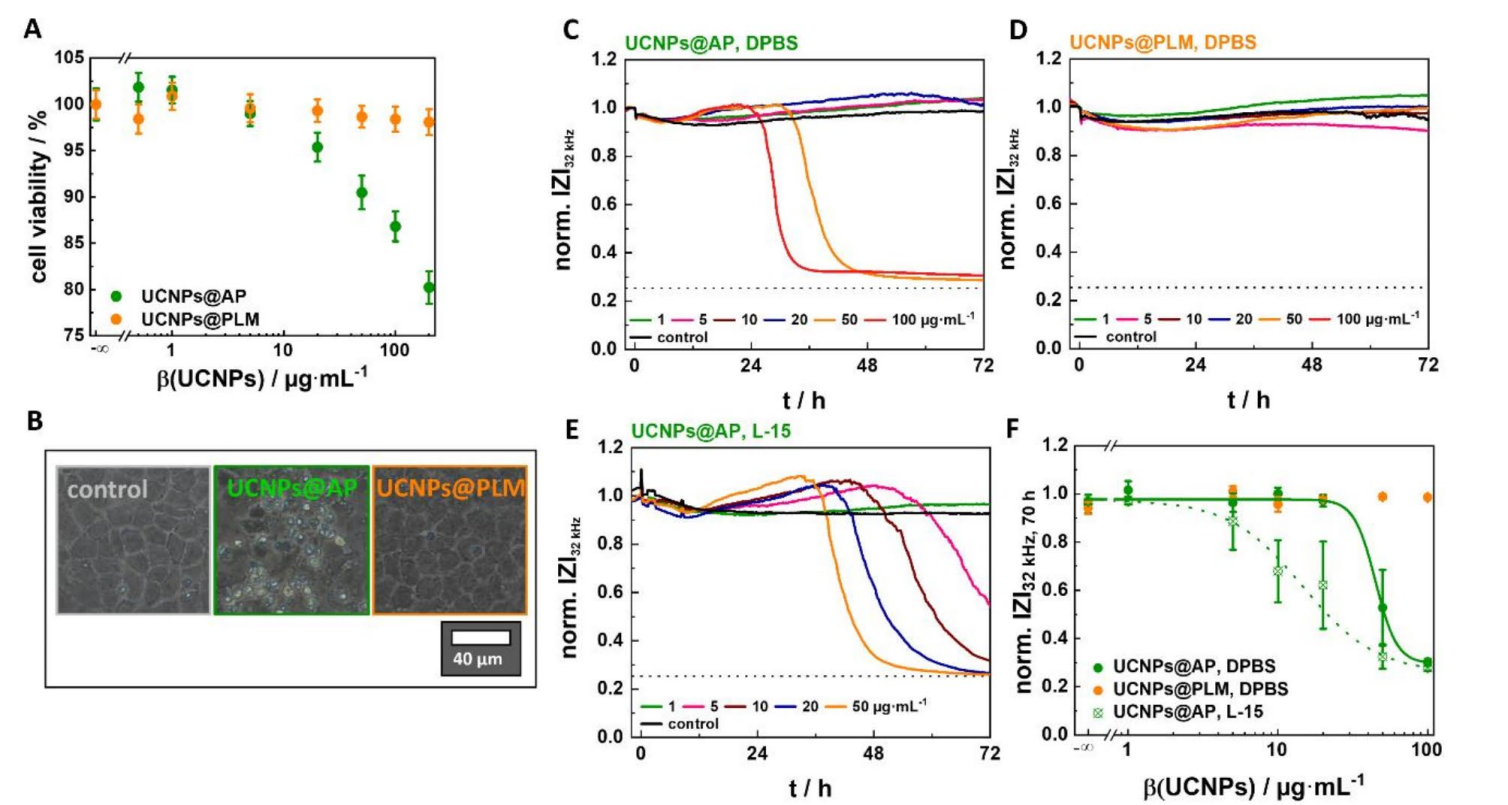
(**A**) Cell viability of NRK cells incubated with 12 nm UCNPS@AP or UCNPs@PLM (mass concentrations (β) of 0.5–200 µg·mL^−1^, L-15, 5% FCS) for 24 h as determined by PrestoBlue assays and (**B**) corresponding phase contrast micrographs (100 µg·mL^−1^). Representative time courses of impedance at 32 kHz of NRK cells, exposed to 33 nm (**C**) UCNPS@AP in DPBS (5% FCS), (**D**) UCNPs@PLM in DPBS (5% FCS), and (**E**) UCNPS@AP in L-15 (5% FCS). Impedances were normalized to the initial value measured at 0 h. The baseline magnitude of impedance was (1.080 ± 0.007) kΩ before particle addition. The dashed line represents the impedance value of a cell-free electrode. (**F**) Concentration-response relationship of the normalized impedance after exposing NRK cells for 70 h to 33 nm UCNPs. EC_50_ values were calculated from the data of three measurements using a 4-parameter logistic model: EC_50_(UCNPs@AP, DPBS, 70 h) = (45 ± 7) µg·mL^−1^ (adjusted R^2^ = 0.999) and EC_50_(UCNPs@AP, L-15, 70 h) = (15 ± 2) µg·mL^−1^ (adjusted R^2^ = 0.999).

Both, the AP and PLM coating, provide UCNPs with sufficient brightness and colloidal stability for bioanalytical applications, but this may change when applied to cells. In order to evaluate the biocompatibility of the UCNPs, non-transformed NRK cells were selected as epithelial-like model cell line from a major organ involved in particle clearance and biodistribution^30^. The UCNPs@AP and UCNPs@PLM were first tested for their cytotoxicity in a resazurin-based PrestoBlue^™^ assay, probing cellular redox metabolism and, thus, cell viability by the cellular content of reduced redox coenzymes. The exposure time was set to the often-used incubation time of 24 h to enable a comparison to data already reported in the literature (Fig. 2A). Cells incubated with the highest concentration of UCNPs@PLM (200 µg·mL^−1^) are still capable of reducing the non-fluorescent resazurin to fluorescent resorufin indicating an intact redox metabolism. In contrast, cells exposed to UCNPs@AP at concentrations > 5 µg·mL^−1^ show a concentration-dependent loss of viability with a ~ 20% drop for the highest concentration (200 µg·mL^−1^). Low concentrations of UCNPs@AP (0.5–1 µg·mL^−1^) increase cell metabolism, resulting in cell viabilities that are formally higher than observed for controls but may indicate the onset of cellular defense mechanisms. This effect, often observed for potentially toxic substances, is defined as *hormesis* and has been already reported for nanoparticles^31–33^. Phase contrast micrographs of cells (Fig. 2B) exposed to UCNPs@PLM are indistinguishable from those of untreated cells. In contrast, micrographs of cell layers treated with UCNPs@AP reveal partly spherical and detached cells as well as cell debris. To determine whether UCNPs@PLM are just not internalized at all or are non-toxic within the 24-hour incubation period, 70 nm ultrathin slices were prepared from NRK cells for electron microscopy analysis after 1–24 h incubation with UCNPs@PLM. A representative overview image (Figure S3 A, 24 h) shows nearly an entire cell with distinguishable compartments. After 1 h, the UCNPs@PLM were observed as individual particles or agglomerates in early and late endosomes (EE, LE) (Figure S3). After 24 h incubation, they were primarily found as aggregates in matured vesicles, such as multivesicular bodies (MVB) and endo-lysosomes (EL) (Figure S3 B, C). Thus, UCNPs@PLM were clearly internalized after 24 h of incubation but did not significantly affect NRK cells, whereas a concentration-dependent effect of UCNPs@AP on cell viability and morphology were observed. However, the Presto Blue assay only reveals the cytotoxicity after a pre-defined exposure time and does neither reveal the time course of toxicity nor the impact of the particles on cell viability for longer exposure times. Therefore, the ECIS assay was selected as a more comprehensive, time-resolved method to assess the particles’ cytotoxicity from changes in cell morphology, loss of adhesion or membrane rupture. NRK cells were grown to confluence on gold-film electrodes prior to their exposure to the different UCNP preparations and their response was monitored for 72 h. The impedance was recorded at an AC frequency of 32 kHz as it provides the highest sensitivity for cell detachment or membrane rupture^34^. Figure 2C and D show representative time courses of the electrical impedance when the cells were treated with increasing concentrations of UCNPs@AP or UCNPs@PLM in DPBS (5% FCS). The impedance of the cells exposed to UCNPs@PLM or buffer remains at basal values for 72 h, providing no indication of toxicity for these particles. In contrast, the impedance of the cells incubated with UCNPs@AP show a concentration-dependent response profile. Treated with the two highest concentrations (≥ 50 µg·mL^−1^), the impedance of the cells increases slightly after ~ 12 h before it drops after (26–36) h to values close to that of the cell-free electrode (dashed line). Lower concentrations of UCNPs@AP initiated only the signal increase but led to no impedance decrease within the recorded time frame. The impedance drop indicates the loss of membrane integrity or detachment of the cells as a response to the particles^34^. The impact of different measurement buffers on the ECIS-readout was examined for the toxic UCNPs@AP. The same concentration-dependent response profile with a transient increase prior to an impedance drop was recorded for UCNPs@AP in L-15 medium (5% FCS), but morphology changes were initiated by significantly lower concentrations of UCNPs@AP (≥ 5 µg·mL^−1^) as compared to experiments performed in DPBS (5% FCS). The time courses in both buffers reveal a toxic impact of the UCNPs@AP, which is not of an immediate but rather intermediate nature. For the toxic UCNPs, a fit of the normalized impedance for 70 h exposure time as a function of particle concentration (Fig. 2F) provided an estimated EC_50_ value of (45 ± 7) µg·mL^−1^ and (15 ± 2) µg·mL^−1^ for NRK cells incubated with 33 nm UCNPs@AP in DPBS (5% FCS, 1 mg·mL^−1^ glucose) or in L-15 (5% FCS), respectively.

Differences in toxicity of UCNPs@AP suspended in serum-containing DPBS or L-15 might be caused by variations in the protein corona formed around the particles in the two media, leading to buffer-dependent internalization efficiency and cytotoxicity. A similar effect has already been observed for the DMEM and RPMI media by Pompa et al. for citrate-coated gold nanoparticles on HeLa and U937 cells^35^. The authors showed a reduced protein corona formation in RPMI together with an improved uptake and higher cytotoxicity. Indeed, it is well-known that the uptake efficiency of nanoparticles and consequently their toxicity are reduced in the presence of a protein corona. The behavior of particles usually depends more on the amount of proteins adsorbed to the particle surface than on the nature of those proteins^36–39^. Particle internalization is also triggered in some cell lines by vitamins like folic acid, which are present in several cell culture media but not in DPBS^40^. Additionally, the aggregation behavior of nanoparticles may vary in different media. As a consequence of the abundance of well-dispersed particles, smaller particle clusters, or large particle aggregates in different media, the toxicity may turn out to be media-dependent^41–43^. However, as the UCNPs do not tend to form large aggregates in any medium as revealed by DLS measurements (Figure S 1), variations of the protein corona are more likely responsible for the buffer-dependent toxicity.

The electrical impedance of the cells, which were exposed to high concentrations of UCNPs@AP, increases transiently prior to a monotonic decrease to the values of a cell-free electrode (Fig. 2C, E). This response profile might reflect an initial swelling of the cells, reducing the widths of the intercellular clefts as the major current pathway. For extended exposure time, the impedance time course indicates membrane rupture after cell swelling as the potential cause for the impedance drop for toxic concentrations. An intact membrane is essential for the cells to form a noticeable barrier to current flow. Upon membrane rupture, for instance as a consequence of osmotic misbalance, the dielectric breakdown of the membrane reduces the impedance increasingly. Membrane rupture is one of the hallmarks of necrosis or related modes of cell death^26^. However, cell shrinkage as a consequence of genetically encoded forms of more apoptotic cell death would also reduce the impedance eventually. It is beyond the scope of this study to identify the exact mechanism of cell death, but the impedance data unequivocally indicates that the cells under study lose their viability in a dose- and time-dependent process triggered by the presence of UCNP@AP but not UCNP@PLM. Indeed, such a transient increase in impedance before cell death, induced by nanomaterials, has already been reported for NRK cells exposed to C-Dots^44^, A549 cells treated with CuO or ZnO NPs^45^, and HEPG2 cells incubated with ZnO NPs^46^. This particular impedance profile has been loosely associated with necrosis simply based on its major morphological hallmarks but without any detailed distinction between the different subcategories of necrotic cell death^44^. Both assays demonstrated that in sharp contrast to UCNPs@PLM, UCNPs@AP strongly stress the cells, clearly indicating that surface engineering is the key to success when it comes to the design of UCNPs for biological applications.

### Internalization of UCNPs@AP and UCNPs@PLM

**Fig. 3:**
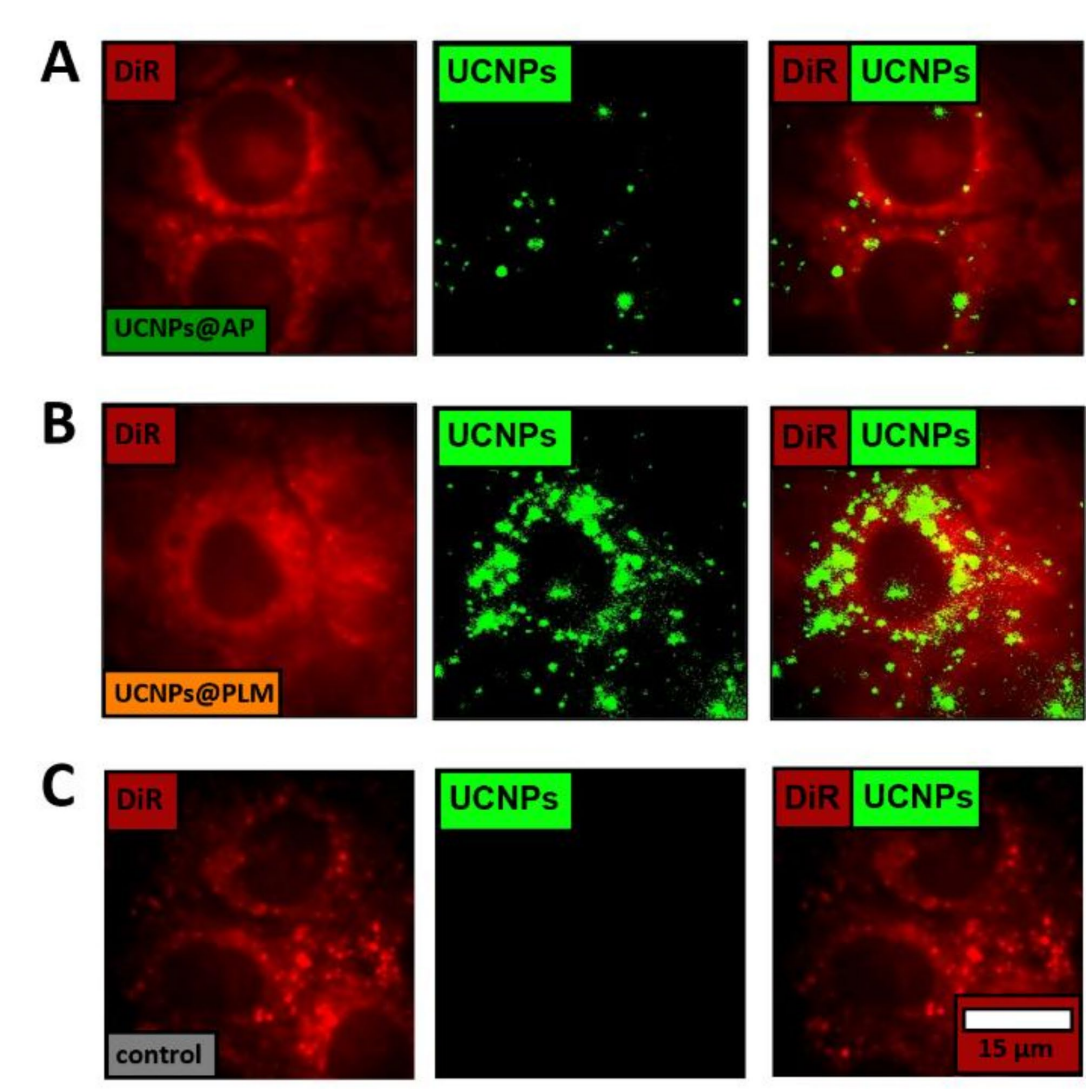
Representative wide-field microscopy images of NRK cells, incubated with 33 nm (**A**) UCNPs@AP and (**B**) UCNPs@PLM (5 µg·mL^−1^) in DPBS (5% FCS, 1 mg·mL^−1^ glucose) and (**C**) no UCNPs as a control for 6 h at 37 °C with green upconversion luminescence (exc.: 974 nm, cw, 8 kW·cm^−2^), red co-staining with DiR (exc.: 633 nm), visualizing the cell bodies and nucleus, and overlay. The mean integrated upconversion intensity was (700 ± 100) cts for cells exposed to UCNPs@AP and (2000 ± 400) cts for cells exposed to UCNPs@PLM (mean ± SEM calculated from two independent experiments, with each experiment comprising 25 regions of interest (ROIs) containing 2–4 cells).

To determine whether the difference in toxicity between UCNPs@AP and UCNPs@PLM was related to a difference in uptake and availability of the two particles in the cytoplasm of NRK cells, we performed (i) an image-based quantification of particle uptake based on UCNP luminescence in individual cells by wide-field upconversion microscopy and (ii) an independent quantification of the total intracellular UCNP content by chemical extraction and subsequent quantification using inductively coupled plasma - mass spectrometry (ICP-MS). The microscopy approach was carried out using the 33 nm UCNPs that were allowed to interact with adherent NRK cells for 6 h. This short incubation time ensured no toxic cell damage and a sufficiently small number of internalized particles to avoid saturation of the detector. Cell bodies and nuclei were visualized using a 1,1’-dioctadecyl-3,3,3’3’-tetramethylindocarbocyanine perchlorate (DiR) co-staining. A representative image of each experimental condition shows upconversion spots in the cells localized around the nucleus (Fig. 3 middle column). The integrated upconversion intensity is higher for the UCNPs@PLM treated cells than for the UCNPs@AP treated ones. Note that in Fig. 3 the color scale is the same for all three experimental conditions and was initially set to accommodate the upconversion of the least bright sample (UCNPs@AP). The mean integrated upconversion intensity has been determined for 25 regions of interest (ROIs with 2–4 cells) from two independent experiments. The mean intensity was (700 ± 100) cts for the cells exposed to UCNPs@AP and (2000 ± 400) cts for those exposed to UCNPs@PLM. Even if one keeps in mind that 33 nm UCNPs@AP are 20% less bright compared to UCNPs@PLM, (Figure S 1E) the upconversion intensity of UCNPs@PLM is significantly higher than that of UCNPs@AP. Therefore, the microscopic studies clearly indicate a reduced uptake of the toxic UCNPs@AP.

The number of internalized UCNPs per cell N_UCNPs_/cell was determined quantitatively by ICP-MS. The cells were incubated with the particles for 24 h as cellular uptake is known to saturate after ~ 24 h^15,47^ and a significant fraction of the cells are still alive within 24 h of UCNP exposure. The cellular uptake was determined to (160 ± 45)·10^3^ UCNPs@AP/cell and (420 ± 90)·10^3^ UCNPs@PLM/cell (24 h, 100 µg·mL^−1^ (12 nm-sized particles), L-15, 5% FCS). Both uptake studies, using independent means of analysis, revealed at least twice as many particles of the non-toxic UCNPs@PLM type in the cell as compared to the toxic UCNPs@AP type. This observation underlines that the amount of internalized UCNPs does not explain the different levels of cytotoxicity.

### Stability of UCNPs@AP and UCNPs@PLM

**Fig. 4:**
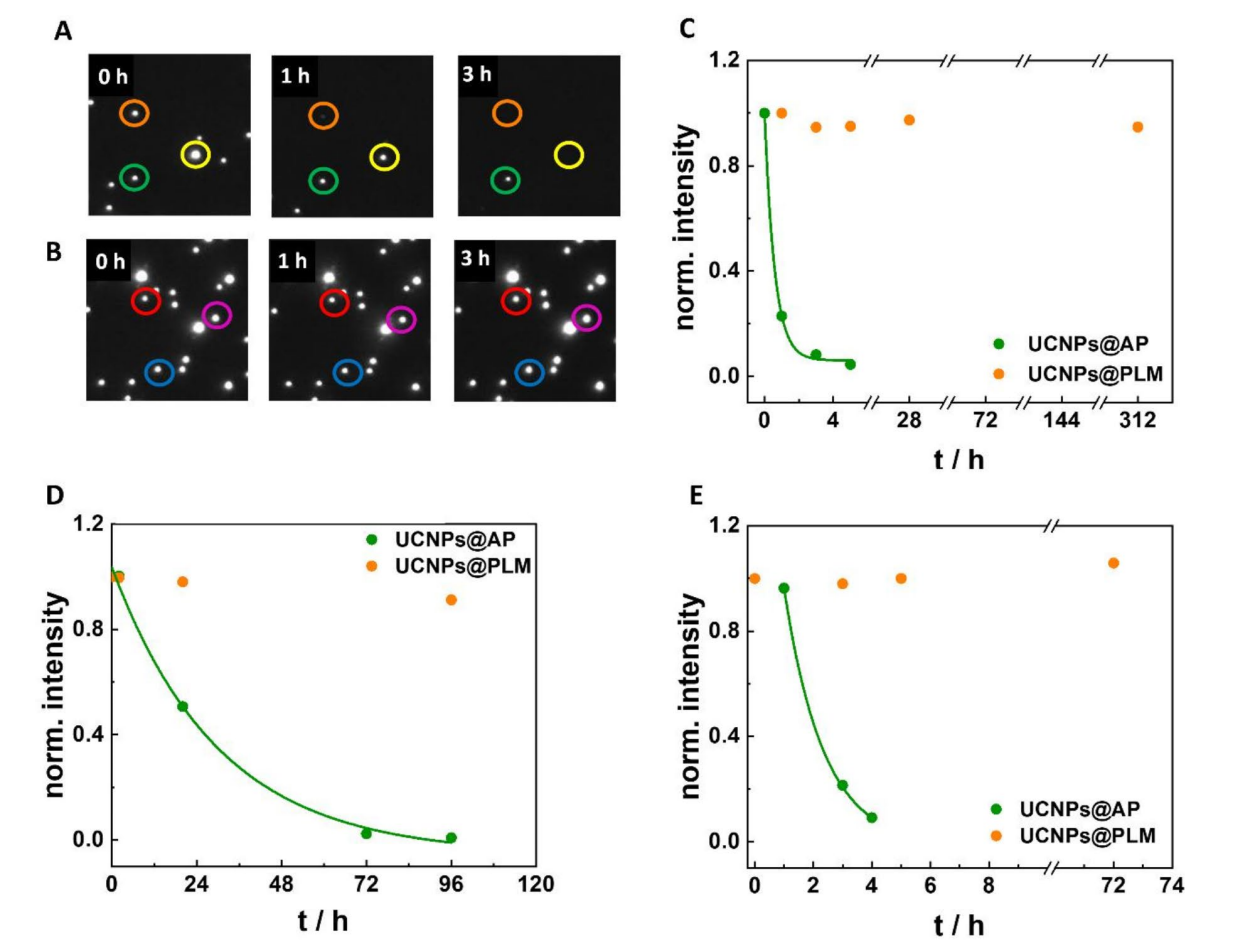
Representative wide-field upconversion micrographs (10 × 10 μm) of 33 nm (**A**) UCNPs@AP and (**B**) UCNPs@PLM in H_2_O at rt at 0 h, 1 h and 3 h upon 974 nm excitation (8 kW·cm^−2^). (**C**) Normalized intensity of UCNPs@AP and UCNPs@PLM in H_2_O at rt for 13 d, (**D**) in H_2_O at rt for 6 d after 30 min pre-incubation with FCS (5%, DPBS) and (**E**) in H_2_O at 37 °C for 3 d after 30 min pre-incubation with FCS (5%, DPBS) with exponential decay fit for UCNPs@AP to estimate dissolution half-lives (t_1/2_). **C**) Experiments were conducted twice. The weighted average of t_1/2_ = (0.41 ± 0.02) h. **D**) Experiments were conducted twice. The weighted average of t_1/2_ = (0.5 ± 0.1) d. **E**) Experiment was conducted just once with t_1/2_= (1.3 ± 0.1) h. The uncertainty corresponds to the uncertainty of the fit. The raw data points correspond to the luminescence intensity of single UCNPs averaged over 100 ROIs covering a total surface of 0.16 mm^2^ (typically *N* = 760 (H_2_O) – 3600 (protein corona) spots).

The chemical stability of UCNPs@AP and UCNPs@PLM was analyzed at the single-particle level by wide-field upconversion microscopy. As demonstrated in a previous study, luminescence loss over time of individual UCNPs in aqueous solution is directly related to ion leakage^8^. Differences in extracellular particle stability provide a direct indicator for the particles’ individual tendency for intracellular ion leakage. The average luminescence intensity of spots attributed to single UCNPs@AP in H_2_O at ambient temperature was observed to completely vanish within less than 5 h (Fig. 3A, B). The time course of the average intensity loss was fitted with an exponential decay function and provided a average luminescence half-live, t_1/2_ (H_2_O, rt), of (0.41 ± 0.02) h (Fig. 4C; weighted average from two datasets). Under the same conditions, the luminescence intensity of the UCNPs@PLM remains constant for at least 13 days. As a biomolecular corona might form around the UCNPs in cellular experiments, the dissolution rate of UCNPs was also studied in FCS-containing medium. The hydrodynamic diameter of the particles in the presence of FCS increased, indicating adsorption of proteins from solution to the particle surface (Figure S1). The average dissolution half-life of UCNPs@AP@FCS, t_1/2_ (FCS, rt), was observed to be (0.5 ± 0.1) d (Fig. 4D; weighted average from two datasets). Compared to the data without FCS incubation, the dissolution half-life was significantly prolonged, suggesting a protecting effect of the protein corona on UCNPs, slowing down particle dissolution. The luminescence intensity of UCNPs@PLM@FCS was stable over the whole duration of the experiment (96 h). Repeating the same experiment at 37 °C, the UCNPs@AP@FCS dissolve faster than at rt with t_1/2_ (FCS, 37 °C) = (1.3 ± 0.1) h indicating that the protecting effect of the FCS coating was reduced at physiological temperature (Fig. 4E; single dataset; uncertainty corresponds to the uncertainty of the fit). Again, the luminescence of UCNPs@PLM@FCS remained constant at 37 °C over the entire observation time (72 h).

According to the data presented in Fig. 4, the protein corona was not sufficient to fully protect the UCNPs@AP from dissolution. Their dissolution was delayed at rt compared to physiological temperature, which is in accordance with studies revealing surface protection by protein corona and an enhanced dissolution at higher temperature^23^. In contrast, PLM-coated particles were perfectly stable under all experimental conditions confirming the very good protecting properties of this surface modification. Similar observations in terms of brightness and colloidal stability in challenging phosphate buffers have already been reported by others^10^. The destabilizing effect of phosphates was also observed within cells. Indeed, the dissolved rare earth ions from NaYF_4_-based UCNPs are known to form precipitating rare earth (RE) phosphates REPO_4_with the organic phosphates in e.g., membrane lipids, nucleotide mono, di, and triphosphates, RNA or DNA. This can even speed up particle disintegration, possibly impair cellular vesicles and induce inflammatory effects^16,18^. The phosphate-enhanced particle disintegration is counteracted by making use of the strong attraction between rare earth ions and phosphates to design an efficient shielding of the particle surface from the media as shown for PLM. Also other phosph(on)ate based ligands as ethylenediaminetetra(methylenephosphonic acid) (EDTMP) or a modified 10-methacryoyl-decylphosphate (HPS) have been proven to enhance the stability of UCNPs NaYF_4_(Yb, Er) under these conditions^16–18^. The different dissolution tendencies of UCNPs@AP and UCNPs@PLM may be observable in dedicated microscopic studies within cells as well.

Additionally, metabolic degradation of the two types of particle-coating bilayers, AP and PLM, may be different within cellular vesicles. The AP coating is rather loose and not all functional groups may be involved in particle stabilization so that the polymer chains partly reach into solution. This may provide an easy access to the amide bonds for non-specific enzymes to induce a stepwise disintegration and pull-off from the particle. In contrast, the PLM coating is of non-polymeric nature and the phospholipids are close to the particle surface. Thus, the functional groups are sterically shielded, protected from enzymatic metabolization, and they may not reach into the active center of proteases or other hydrolases. It seems plausible that individual biotransformation, vesicle membrane damage, and release of vesicle loading into the cytoplasm is the reason for the observed toxicity of poorly shielded particles. The vesicular membrane leakage and release of vesicular enzymes may trigger direct digestion of cellular components and/or the activation of signaling cascades inducing cell death. Similar to these observations, a correlation between chemical stability of particles and cell viability has been reported for UCNPs with non-perfect protective surface coatings, while NaYF_4_(Yb, Er)@EDTMP showed no significant ion leakage or any impact on cell viability of human endothelial cells EA.hy926 and human myeloid cells THP-1^16,18^. The experiments described above support the conclusion that the toxicity of UCNPs@AP, observed for epithelial NRK cells, was evoked by a lower surface shielding of the polymer coating and may be further intensified by metabolic degradation.

## Conclusions

Both, intrinsically non-toxic PLM or AP surface modifications produce UCNPs with colloidal stability and brightness. However, UCNPs@AP and UCNPs@PLM differ significantly with respect to cytotoxicity. Viability and morphology of normal epithelial-like cells were strikingly affected by UCNPs@AP, while UCNPs@PLM did not induce any effect on NRK cells, even though they were internalized in higher amounts as compared to UCNPs@AP. Time resolved impedance analysis of cells revealed a delayed toxicity of the UCNPs@AP with an onset of cell death after around 30 h of exposure. The response profile shows a transient cell swelling prior to membrane permeabilization upon exposure to poorly shielded UCNPs@AP. Based on our data, the difference in toxicity is supposedly caused by a difference in chemical stability of the particles. UCNP@PLM did neither disintegrate at room temperature in water nor in presence of serum at physiological temperatures. In contrast, UCNPs@AP dissolved under all conditions within hours. Their partial disintegration most likely also happens within the cells. Ion leakage from poorly shielded UCNPs@AP and their toxic effect might contribute to cell death. This study clearly demonstrates that a toxicity analysis by common cell viability assays is not sufficient to provide a comprehensive picture of UCNPs biocompatibility. Microscopic analysis together with electric cell-substrate impedance sensing revealed different interactions of epithelial cells with UCNPs, that was clearly dependent on the surface coating, leading to both, internalization of individual and agglomerated UCNPs. These findings stress out the importance of protective surface coatings on nanoparticle fate and interactions within the cell and might be helpful for surface engineering of inorganic nanoparticles suitable for long time applications in biological systems. The PLM coating with freely available amine groups is also a good choice for surface functionalization e.g. by EDC/NHS activation or by click chemistry.

## Methods

### Particle synthesis

The syntheses of the core-shell particles NaYF_4_(Yb, Er)@NaYF_4_ were carried out by the stepwise injection of shell precursor particles to a boiling suspension of ~ 10 nm or ~ 25 nm core particles of NaYF_4_(20%Yb,2%Er) in a boiling mixture of 1-octadecene and oleic acid as solvents, as reported previously^10,28,29^.

### Surface functionalization

The amphiphilic polymer (AP) coated particles (UCNPs@AP) were prepared from poly(isobutyl-maleic anhydride) (PIBMAD) with 75% dodecylamine side chains. The phospholipid membrane (PLM) coated particles were obtained by wrapping the UCNPs with a lipid mixture, consisting of 1,2-dioleoyl-sn-glycero-3-phosphate (DOPA, 64%), 1,2-dioleoyl-sn-glycero-3-phosphoethanolamine (DOPE, 7%) and cholesterol (29%). The detailed procedures were published recently^10^.

### Preparation of amphiphilic polymer agglomerates without UCNP core

Amphiphilic polymer agglomerates were prepared following the same protocol as for UCNPs@AP without using UCNPs and by omitting the centrifugation steps. The AP nanostructures were diluted 1:10 (finally 15 mM monomer concentration) according to the usual dilution of UCNPs@AP to 100 µg·mL^−1^.

### Single particle luminescence measurements

Single particle luminescence was measured by a home-built wide-field upconversion microscope described in detail in our previous work^8^. Briefly, an inverted microscope IX71 (Olympus, Japan) equipped with a high numerical aperture objective (Olympus, UApo N 100×/1.49 oil) was coupled to a 974 nm single mode fiber laser (CW, 350 mW, Qphotonics, USA) to excite the UCNPs with an excitation power density of 8 kW·cm^−2^ in HILO illumination. Laser excitation was filtered by a long-pass excitation filter (ET780LP, Chroma). Luminescence emission was separated from the excitation beam by using a short pass dichroic mirror (T875spxrxt, Chroma), while the residual laser light was removed by a low pass filter (E700SP, Chroma). Emission was detected by an electron multiplying CCD camera (Hamamatsu, ImagEM X2 C9100-23B) with a 100 ms exposure time. Acquisition was fully automated and controlled by scripts within the MicroManager framework and image analysis was performed with Matlab (MathWorks). For particle stability experiments, UCNPs@AP and UCNPs@PLM in H_2_O or in DPBS (5% FCS, 1 mg·mL^−1^ glucose) were immobilized on a polyethyleneimine (PEI) coated 8 well chambered cover glass from Cellvis for 30 min (0.6 mL, 0.2 µg·mL^−1^ per well). The particle suspension was supplemented with NaF (1 mM) at rt or 37 °C to prevent possible particle dissolution during sample preparation and thus allowed to measure the luminescence of intact particles. After imaging the initial upconversion luminescence intensity in NaF-containing solution at t = 0 h, the adsorbed UCNPs were washed three times and kept under H_2_O at rt or 37 °C for the following measurements. It was ensured that UCNPs did not detach during the washing procedure.

All images (ROI 40 μm x 40 μm) were obtained as an averaged stack of 100 images and drift-corrected before the intensity of luminescence spots attributed to single UCNPs was determined by a custom Matlab script. The UCNPs spot intensities extracted from 100 ROIs were plotted in a histogram and fitted with a gaussian model to obtain the mean intensity (Figure S 1). The total intensity of all integrated luminescence spots was determined for each time point and normalized to the initial value in order to monitor the luminescence over the time course of the experiment. In the case of luminescence losses, the data were fitted with a mono exponential decay function.

### Cell experiments

Normal rat kidney (NRK-52E; Leibniz Institute DSMZ GmbH, Germany) cells (subculture 14–29) were seeded on the substrates to confluence (250 000 cells·cm^−2^) in culture medium (DMEM (3.7 g NaHCO_3_, 4.5 g·L^−1^ D-glucose) 5% (v/v) FCS, 1 mM L-glutamine, 100 µg·mL^−1^ penicillin and 100 µg·mL^−1^ streptomycin). They were cultivated at 37 °C and 5% CO_2_ for two days with a fresh medium supply after 24 h.

### Cell viability assay PrestoBlue

NRK cells were seeded on 96 well plates. After two days of cultivation, the 12 nm UCNPs@AP and UCNPs@PLM were added to the wells (100 µL, 0.5–200 µg·mL^−1^ in Leibovitz L-15 medium + 5% FCS + 100 µg·mL − 1 penicillin and 100 µg·mL 1 streptomycin) and incubated at 37 °C and 0% CO_2_ for 24 h. After aspiration of the particle solutions, the cells were incubated with the resazurin solution (100 µL, 10 µg·mL^−1^, DPBS + 1 mg·mL^−1^ glucose) at 37 °C and 0% CO_2_ for 60 min. The fluorescence intensity was measured at λ_em_ = 600 nm (exc.: 532 nm) with the microplate reader GENios (Tecan, Switzlerland). Cells exposed to Triton X-100 (0.5%, v/v) served as positive (zero viability) control. The conversion of resazurin by UCNPs was excluded in control measurements with UCNPs and resazurin but without cells. The PrestoBlue assay was performed three times each with a three-fold replication for every condition to calculate the instrumental weighted mean with the corresponding error.

### Electric cell-substrate impedance sensing (ECIS)

NRK cells were seeded on 8W10E arrays (Applied BioPhysics Inc., USA). After two days of cultivation, the impedance measurement was started in L-15 (5% FCS, 100 µg·mL^−1^ penicillin, 100 µg·mL^−1^ streptomycin) or DPBS (5% FCS, 1 mg·mL^−1^ glucose) at 37 °C, 0% or 5% CO_2_, recorded by the ECIS Z-Θ device (Applied BioPhysics Inc.). After reaching a stable baseline, the 33 nm UCNPs@PLM or UCNPs@AP were added (200 µL, final concentrations 1–100 µg·mL^−1^) and the impedance was continuously monitored for at least 70 h. Measurements were repeated at least three times. The impedance time courses at 32 kHz were normalized to the last time point before particle addition (|Z|/|Z|0 h, time point 0 h). The normalized impedance after 70 h was averaged for each condition (mean ± standard error of mean (SEM)) and correlated to the particle concentration. Concentration dependent response was approximated by a four parametric logistic model.

### Electron microscopy

NRK cells were seeded on 12 mm coverslips in 24 well arrays (37 °C, 5% CO_2_). After 48 h the culture medium was replaced by the 12–33 nm UCNPs@PLM suspension (600 µL 50 µg·mL^−1^ of 12 nm UCNPs@PLM or 200 µg·mL^−1^ of 33 nm UCNPs@PLM in culture medium). The cells were incubated with the particles for 1–24 h at 37 °C and 5% CO_2_, washed twice with DPBS and fixed with glutaraldehyde (2%, w/v, 0.1 M cacodylate buffer, pH 7.4) for 3 min. Cells were washed 3 × 2 min with cacodylate buffer (0.1 M, pH 7.4) and 3 × 2 min with H_2_O prior to the incubation with uranyl acetate (1%, w/v, H_2_O) on ice for 60 min. The samples were washed 5 × 2 min with H_2_O before dehydration with ethanol in rising concentrations (30–100%, each 2 × 2 min) and acetone (2 × 2 min). They were kept in a mixture of epon and acetone (1:1) for 5 min before hardening in epon at 30 °C for 1 h and at 60 °C for 2–3 h. Cover slips were blasted with liquid nitrogen. The 70 nm ultrathin sections were trimmed and cut with a diamond knife set on the microtome UC6 (Leica). Micrographs of the cell sections were taken with a transmission electron microscope (Zeiss 902) at 80 kV. All samples were prepared twice.

### Wide-field upconversion microscopy and inhibition study

NRK cells were seeded in glass rings (d = 6 mm), glued in µ-dishes with glass bottom purchased from ibidi GmbH (Gräfelfing, Germany). After 48 h cells were treated with the dye 1,1-dioctadecyl-3,3,3,3-tetramethylindotricarbocyanine iodide (DiR, 2.5 µM in DPBS + 5% FCS, 1 mg·mL^−1^ glucose) for 20 min at 37 °C and 0% CO_2_. The dye solution for co-staining was removed and the cells were washed twice with DPBS before particle suspensions of 33 nm UCNPs@AP or UCNPs@PLM (10 µg·mL^−1^) in DPBS (5% FCS, 1 mg·mL^−1^ glucose) were added for 1–6 h at 37 °C and 0% CO_2_. For the inhibition study, NRK cells were incubated with UCNPs@PLM at 4 °C, in presence of dynasore (200 µM) or sucrose (0.45 M) and UCNPs@PLM (5 µg·mL^−1^) in DPBS (5% FCS, 1 mg·mL^−1^ glucose) at 37 °C. After incubation, the cells were washed twice with DPBS and incubated with paraformaldehyde (4%, w/v, DPBS) at room temperature for 10 min. Finally, the cells were washed three times with DPBS. Separate microscopic stacks were taken upon 974 nm (8 kW·cm^−2^) and 633 nm irradiation with the aforementioned wide-field microscope, additionally equipped with a 633 nm laser line (LGK 7665 P18, LASOS, Germany), dichroic mirror (Di01-R635-25 × 36, Semrock), notch filter (Stop Line Notch Filter 633, Semrock) and a long pass filter (647 LP Edge Basic edge basic, Semrock). The mean upconversion intensity integrated over one cell has been determined for 25 regions of interest (ROIs with typically 2–4 cells) for two independent experiments with imageJ. The mean intensity/ROI was depicted in a box plot, showing the mean ± SEM, the median and 25–75% of the data points in the boxes.

### ICP-MS

Normal rat kidney (NRK) cells were seeded on 96 well plates. After 48 h the cells were exposed to 12 nm UCNPs@AP and UCNPs@PLM (100 µg·mL^−1^) in L-15 (5% FCS, 100 µg·mL^−1^ penicillin, 100 µg·mL^−1^ streptomycin) at 37 °C and 0% CO_2_ for 24 h. The cells were washed twice with DPBS and incubated with trypsin (0.05% (w/v) with 1 mM EDTA in PBS, 1 mL) for 30 min before they were dried at 70 °C. The cell samples were suspended with H_2_SO_4_ (≥ 95%, w/w, 0.5 mL) for 15 min, diluted with H_2_O (9.5 mL), equipped with Rh standard (4 µL, 10 000 ppb, Perkin Elmer) and filtered (200 nm, polyether sulfone) prior to the analysis. The amount of rare earth ions was used to determine the number of particles N_UCNPs_. The mean number of cells per well was obtained with a Bürker hemacytometer after cell removal from the surface to calculate a mean number of particles per cell N_UCNPs/cell_. The experiment was repeated two individual times with a two-fold determination and the mean ± SEM was calculated.

## Electronic Supplementary Material

Below is the link to the electronic supplementary material.

Supplementary Material 1

## Data Availability

The datasets used and/or analysed during the current study are available from the corresponding author on reasonable request.

## Animal experiments statement

There were no animals involved in our study submitted to Scientific Reports. It is an entirely in vitro approach using cultured cell lines exclusively.

## References

[1] All, A. H. et al. Expanding the Toolbox of Upconversion Nanoparticles for In Vivo Optogenetics and Neuromodulation. *Adv. Mater.* **31**, e1803474. 10.1002/adma.201803474 (2019). 31432555 10.1002/adma.201803474

[2] Chen, B. & Wang, F. Emerging Frontiers of Upconversion Nanoparticles. *Trends Chem.* **2**, 427–439. 10.1016/j.trechm.2020.01.008 (2020).

[3] Del Rosal, B. & Jaque, D. Upconversion nanoparticles for in vivo applications: limitations and future perspectives. *Methods Appl. Fluoresc*. **7**, 22001. 10.1088/2050-6120/ab029f (2019). 10.1088/2050-6120/ab029f 30695767

[4] Zhang, Y., Zhu, X. & Zhang, Y. Exploring Heterostructured Upconversion Nanoparticles: From Rational Engineering to Diverse Applications. *ACS nano*. **15**, 3709–3735. 10.1021/acsnano.0c09231 (2021). 10.1021/acsnano.0c09231 33689307

[5] Haase, M. & Schäfer, H. Upconverting nanoparticles. *Angew Chem. Int. Ed.* **50**, 5808–5829. 10.1002/anie.201005159 (2011). 10.1002/anie.201005159 21626614

[6] Yang, Y. Upconversion nanophosphors for use in bioimaging, therapy, drug delivery and bioassays. *Microchim. Acta*. **181**, 263–294. 10.1007/s00604-013-1139-8 (2014).

[7] Li, R. et al. Surface interactions with compartmentalized cellular phosphates explain rare earth oxide nanoparticle hazard and provide opportunities for safer design. *ACS nano*. **8**, 1771–1783. 10.1021/nn406166n (2014). 24417322 10.1021/nn406166n PMC3988685

[8] Dukhno, O. et al. Time-dependent luminescence loss for individual upconversion nanoparticles upon dilution in aqueous solution. *Nanoscale* **10**, 15904–15910. 10.1039/C8NR03892A (2018). 30106079 10.1039/c8nr03892a

[9] Lahtinen, S. et al. Disintegration of Hexagonal NaYF 4:Yb 3+, Er 3 + Upconverting Nanoparticles in Aqueous Media: The Role of Fluoride in Solubility Equilibrium. *J. Phys. Chem. C*. **121**, 656–665. 10.1021/acs.jpcc.6b09301 (2017).

[10] Märkl, S., Schroter, A. & Hirsch, T. Small and Bright Water-Protected Upconversion Nanoparticles with Long-Time Stability in Complex, Aqueous Media by Phospholipid Membrane Coating. *Nano Lett.* **20**, 8620–8625. 10.1021/acs.nanolett.0c03327 (2020). 33164510 10.1021/acs.nanolett.0c03327

[11] Yang, W., Wang, L., Mettenbrink, E. M., DeAngelis, P. L. & Wilhelm, S. Nanoparticle Toxicology. *Annu. Rev. Pharmacol. Toxicol.* **61**, 269–289. 10.1146/annurev-pharmtox-032320-110338 (2021). 32841092 10.1146/annurev-pharmtox-032320-110338

[12] Torresan, M. F. & Wolosiuk, A. Critical Aspects on the Chemical Stability of NaYF4-Based Upconverting Nanoparticles for Biomedical Applications. *ACS Appl. Bio Mater.* **4**, 1191–1210. 10.1021/acsabm.0c01562 (2021). 35014473 10.1021/acsabm.0c01562

[13] Guller, A. E. et al. Cytotoxicity and non-specific cellular uptake of bare and surface-modified upconversion nanoparticles in human skin cells. *Nano Res.* **8**, 1546–1562. 10.1007/s12274-014-0641-6 (2015).

[14] Gnach, A., Lipinski, T., Bednarkiewicz, A., Rybka, J. & Capobianco, J. A. Upconverting nanoparticles: assessing the toxicity. *Chem. Soc. Rev.* **44**, 1561–1584. 10.1039/C4CS00177J (2015). 25176037 10.1039/c4cs00177j

[15] Wang, C., He, M., Chen, B. & Hu, B. Study on cytotoxicity, cellular uptake and elimination of rare-earth-doped upconversion nanoparticles in human hepatocellular carcinoma cells. *Ecotoxicol. Environ. Saf.* **203**, 110951. 10.1016/j.ecoenv.2020.110951 (2020). 32678752 10.1016/j.ecoenv.2020.110951

[16] Li, R. et al. Enhancing the imaging and biosafety of upconversion nanoparticles through phosphonate coating. *ACS nano*. **9**, 3293–3306. 10.1021/acsnano.5b00439 (2015). 25727446 10.1021/acsnano.5b00439 PMC4415359

[17] Mendez-Gonzalez, D. et al. Upconverting Nanoparticles in Aqueous Media: Not a Dead-End Road. Avoiding Degradation by Using Hydrophobic Polymer Shells. *Small* **18**, e2105652. 10.1002/smll.202105652 (2022). 34897995 10.1002/smll.202105652

[18] Lisjak, D. et al. NaYF4-based upconverting nanoparticles with optimized phosphonate coatings for chemical stability and viability of human endothelial cells. *Methods Appl. Fluoresc*. **10**10.1088/2050-6120/ac41ba (2021). 10.1088/2050-6120/ac41ba 34883469

[19] Vozlič, M. et al. Formation of phosphonate coatings for improved chemical stability of upconverting nanoparticles under physiological conditions. *Dalton Trans.* **50**, 6588–6597. 10.1039/D1DT00304F (2021). 10.1039/d1dt00304f 33899872

[20] Plohl, O. et al. Amphiphilic coatings for the protection of upconverting nanoparticles against dissolution in aqueous media. *Dalton Trans.* **46**, 6975–6984. 10.1039/C7DT00529F (2017). 28513723 10.1039/c7dt00529f

[21] Kembuan, C., Oliveira, H. & Graf, C. Effect of different silica coatings on the toxicity of upconversion nanoparticles on RAW 264.7 macrophage cells. *Beilstein J. Nanotechnol*. **12**, 35–48. 10.3762/bjnano.12.3 (2021). 33489665 10.3762/bjnano.12.3 PMC7801781

[22] Estebanez, N., González-Béjar, M. & Pérez-Prieto, J. Polysulfonate Cappings on Upconversion Nanoparticles Prevent Their Disintegration in Water and Provide Superior Stability in a Highly Acidic Medium. *ACS omega*. **4**, 3012–3019. 10.1021/acsomega.8b03015 (2019). 31459525 10.1021/acsomega.8b03015 PMC6648593

[23] Saleh, M. I. et al. Assessing the protective effects of different surface coatings on NaYF4:Yb3+, Er3 + upconverting nanoparticles in buffer and DMEM. *Sci. Rep.* **10**10.1038/s41598-020-76116-z (2020). 10.1038/s41598-020-76116-z PMC7652843 33168848

[24] Bastos, V. et al. Stability, dissolution, and cytotoxicity of NaYF4-upconversion nanoparticles with different coatings. *Sci. Rep.* **12**10.1038/s41598-022-07630-5 (2022). 10.1038/s41598-022-07630-5 PMC8904531 35260656

[25] Giaever, I. & Keese, C. R. Monitoring fibroblast behavior in tissue culture with an applied electric field. *Proc. Natl. Acad. Sci. U.S.A.* **81**, 3761–3764 (1984). 10.1073/pnas.81.12.3761 10.1073/pnas.81.12.3761 PMC345299 6587391

[26] Zinkl, M. & Wegener, J. Using animal cells as sensors for xenobiotics: monitoring phenotypic changes by multimodal impedance assays. *Curr. Opin. Environ. Sci.* **10**, 30–37. 10.1016/j.coesh.2019.08.007 (2019).

[27] Oliveira, H. et al. Critical Considerations on the Clinical Translation of Upconversion Nanoparticles (UCNPs): Recommendations from the European Upconversion Network (COST Action CM1403). *Adv. Healthc. Mater.* **8**, e1801233. 10.1002/adhm.201801233 (2019). 30536962 10.1002/adhm.201801233

[28] Muhr, V. et al. Particle-Size-Dependent Förster Resonance Energy Transfer from Upconversion Nanoparticles to Organic Dyes. *Anal. Chem.* **89**, 4868–4874. 10.1021/acs.analchem.6b04662 (2017). 28325045 10.1021/acs.analchem.6b04662

[29] Wilhelm, S. et al. Water dispersible upconverting nanoparticles: effects of surface modification on their luminescence and colloidal stability. *Nanoscale* **7**, 1403–1410. 10.1039/C4NR05954A (2015). 25503253 10.1039/c4nr05954a

[30] Du, B., Yu, M. & Zheng, J. Transport and interactions of nanoparticles in the kidneys. *Nat. Rev. Mater.* **3**, 358–374. 10.1038/s41578-018-0038-3 (2018).

[31] Iavicoli, I., Calabrese, E. J. & Nascarella, M. A. Exposure to nanoparticles and hormesis. *Dose-Response* **8**, 501–517. 10.2203/dose-response.10-016.Iavicoli (2010). 10.2203/dose-response.10-016.Iavicoli PMC2990066 21191487

[32] Jiao, Z. H. et al. Hormesis effects of silver nanoparticles at non-cytotoxic doses to human hepatoma cells. *PloS one*. **9**, e102564. 10.1371/journal.pone.0102564 (2014). 10.1371/journal.pone.0102564 PMC4102499 25033410

[33] Stebbing, A. Hormesis — The stimulation of growth by low levels of inhibitors. *Sci. Total Environ.* **22**, 213–234. 10.1016/0048-9697(82)90066-3 (1982). 7043732 10.1016/0048-9697(82)90066-3

[34] Lukic, S. & Wegener, J. Impedimetric Monitoring of Cell-Based Assays. *eLS* **1-8**10.1002/9780470015902.a0025710 (2015).

[35] Maiorano, G. et al. Effects of cell culture media on the dynamic formation of protein-nanoparticle complexes and influence on the cellular response. *ACS nano*. **4**, 7481–7491. 10.1021/nn101557e (2010). 21082814 10.1021/nn101557e

[36] Chandran, P., Riviere, J. E. & Monteiro-Riviere, N. A. Surface chemistry of gold nanoparticles determines the biocorona composition impacting cellular uptake, toxicity and gene expression profiles in human endothelial cells. *Nanotoxicology* **11**, 507–519. 10.1080/17435390.2017.1314036 (2017). 28420299 10.1080/17435390.2017.1314036

[37] Cheng, X. et al. Protein Corona Influences Cellular Uptake of Gold Nanoparticles by Phagocytic and Nonphagocytic Cells in a Size-Dependent Manner. *ACS Appl. Mater. Interfaces*. **7**, 20568–20575. 10.1021/acsami.5b04290 (2015). 26364560 10.1021/acsami.5b04290

[38] Kelly, P. M. et al. Mapping protein binding sites on the biomolecular corona of nanoparticles. *Nat. Nanotechnol*. **10**, 472–479. 10.1038/nnano.2015.47 (2015). 25822932 10.1038/nnano.2015.47

[39] Lesniak, A. et al. Effects of the presence or absence of a protein corona on silica nanoparticle uptake and impact on cells. *ACS nano*. **6**, 5845–5857. 10.1021/nn300223w (2012). 22721453 10.1021/nn300223w

[40] de Sousa, M. et al. Understanding nanoparticle endocytosis to improve targeting strategies in nanomedicine. *Chem. Soc. Rev.* **50**, 5397–5434. 10.1039/D0CS01127D (2021). 33666625 10.1039/d0cs01127d PMC8111542

[41] Buford, M. C., Hamilton, R. F. & Holian, A. A comparison of dispersing media for various engineered carbon nanoparticles. *Part. Fibre Toxicol.* **4**, 6 (2007). 10.1186/1743-8977-4-6 10.1186/1743-8977-4-6 PMC1950524 17655771

[42] DeLoid, G. M., Cohen, J. M., Pyrgiotakis, G. & Demokritou, P. Preparation, characterization, and in vitro dosimetry of dispersed, engineered nanomaterials. *Nat. Protoc.* **12**, 355–371. 10.1038/nprot.2016.172 (2017). 28102836 10.1038/nprot.2016.172 PMC5857388

[43] Feliu, N., Sun, X., Alvarez Puebla, R. A. & Parak, W. J. Quantitative Particle-Cell Interaction: Some Basic Physicochemical Pitfalls. *Langmuir* **33**, 6639–6646. 10.1021/acs.langmuir.6b04629 (2017). 28379704 10.1021/acs.langmuir.6b04629

[44] Sperber, M. et al. (eds) *Monitoring the Impact of Nanomaterials on Animal Cells by Impedance Analysis: A Noninvasive, Label-Free, and Multimodal Approach* (Springer International Publishing, 2016).

[45] Seiffert, J. M. et al. Dynamic monitoring of metal oxide nanoparticle toxicity by label free impedance sensing. *Chem. Res. Toxicol.* **25**, 140–152. 10.1021/tx200355m (2012). 22054034 10.1021/tx200355m

[46] Bartczak, D., Baradez, M. O., Goenaga-Infante, H. & Marshall, D. Label-free monitoring of the nanoparticle surface modification effects on cellular uptake, trafficking and toxicity. *Toxicol. Res.* **4**, 169–176. 10.1039/c4tx00105b (2015).

[47] Rojas-Gutierrez, P. A., Bekah, D., Seuntjens, J. & DeWolf, C. Capobianco. Cellular Uptake, Cytotoxicity and Trafficking of Supported Lipid-Bilayer-Coated Lanthanide Upconverting Nanoparticles in Alveolar Lung Cancer Cells. *ACS Appl. Bio Mater.* **2**, 4527–4536. 10.1021/acsabm.9b00649 (2019). 35021412 10.1021/acsabm.9b00649

