# Supplementary material for: Impact of surface chemistry of upconversion nanoparticles on time-dependent cytotoxicity in non-cancerous epithelial cells
Source: Sci Rep. 2024 Dec 23;14:30610. doi: 10.1038/s41598-024-83406-3 (PMC11666596; doi:10.1038/s41598-024-83406-3)
Supplement: Supplementary file 1 — Supplementary Material 1 [file 41598_2024_83406_MOESM1_ESM.docx]

Supporting Information

**Impact of the Surface Chemistry of Upconversion Nanoparticles on Time-dependent Cytotoxicity in Non-cancerous Epithelial Cells**

Susanne Märkl,^a^ Frederic Przybilla,^b^ Reinhard Rachel,^c^ Thomas Hirsch,^a^ Max Keller,^d^

Ralph Witzgall,^e^ Yves Mely,^b^ Joachim Wegener*^a,f^

^a^ University of Regensburg, Institute for Analytical Chemistry, Chemo- and Biosensors, 93053 Regensburg, Germany.

^b^ Laboratory of Biomaging and Pathologies, UMR 7021 CNRS, University of Strasbourg, 67000 Strasbourg, France.

^c^ University of Regensburg, Centre for EM, 93053 Regensburg, Germany.

^d^ University of Regensburg, Institute for Pharmacy, 93053 Regensburg, Germany.

^e^ University of Regensburg, Institute for Anatomy, 93053 Regensburg, Germany.

^f^ Fraunhofer Research Institution for Microsystems and Solid State Technology EMFT, 93053 Regensburg, Germany.

Table of Contents

[Chemicals 3](#_Toc102322962)

[Methods of Particle Characterization 3](#_Toc102322963)

[Particle Preparation and Characterization 5](#_Toc102322964)

[Supplementary Figures and Tables 7](#_Toc102322965)

[References 10](#_Toc102322966)

# Chemicals

Hexane (95%) was bought from Acros. The chemicals 1-octadecene (90%) and oleic acid (90%) were purchased from Alfa Aesar. 1,2-Dioleoyl-*sn*-glycero-3-phosphate (sodium salt) (DOPA) and 1,2-dioleoyl-sn-glycero-3-phosphoethanolamine (DOPE) were bought from Avanti Polar Lipids. Multi element standard (Y^3+^, Yb^3+,^ Er^3+^, Tm^3+^, Gd^3+^) for ICP-OES measurements was bought from Bernd Kraft. CaCl_2_ (94%) was from Carl Roth. Normal rat kidney epithelial cells of the cell line NRK-52E were obtained from Deutsche Sammlung von Mikroorganismen und Zellkulturen. Chloroform (99.98 %), DMF (99.9%) and methanol (99.99%) were from Fisher Chemical. Multi element standard (pure plus) and Rh standard for ICP-MS measurements were obtained from PerkinElmer. Fetal bovine serum was from Gibco. Dynasore (99%) was from Selleckchem. Cholesterol (99%), dodecylamine (98%), Dulbecco´s phosphate buffered saline (PBS), Dulbecco’s modified eagle’s medium (DMEM) - high glucose, EDTA in DPBS^--^, erbium(III) chloride, D-glucose, L-glutamine, Leibovitz L-15 medium, Na-oleate (82%), NOBF_4_ (95%), penicillin/streptomycin, poly(acrylic acid) (2,100 kDa), poly(isobutylene-alt-maleic anhydride (6,000 kDa), sucrose, resazurin, Triton X-100 and trypsin - EDTA were purchased from Sigma Aldrich. Ytterbium(III) chloride hexahydrate (99.999%) and yttrium(III) chloride hexahydrate (99.99%) were obtained from Treibacher Industrie AG. Cyclohexane (100%) and NaCl was from VWR Chemicals. All chemicals were used as received without any further purification. All aqueous solutions were prepared with double distilled water.

# Methods of Particle Characterization

The composition of the UCNPs and their mass concentrations were determined by optical emission spectroscopy combined with inductively coupled plasma excitation (ICP-OES) Spectroblue FMX36 from Spectro. Calibrations were performed using a multielement standard from Bernd Kraft. The UCNPs were dried, dissolved in sulfuric acid (500 µL, ≥ 95%, w/w) and diluted with HNO_3_ (9.5 mL, 1.5 M).

Luminescence spectra were recorded with a home-built set-up. The spectrometer (225 – 1000 nm) and the Software Waves were obtained from former RGB photonics. The 980 nm, 200 mW (continuous wave, cw) laser module was obtained from Picotronic. Measurements in aqueous media were performed at 85 W·cm^‑2^.

Dynamic light scattering measurements of the particles (approx. 0.4 mg·mL^‑1^) were performed with a Malvern Zetasizer Nano ZS. All size distributions are intensity weighted and the mean particle diameter with the corresponding standard deviations (SD) results from three measurements.

The surface zeta potential of the UCNPs@PLM (approx. 0.2 mg·mL^‑1^) in NaCl (10 mM) was determined with the Malvern Zetasizer Nano ZS. The average zeta potential and its standard deviation have been obtained from three measurements.

Transmission electron microscopy (TEM) was performed with the 120 kV CM12 microscope from Phillips. The particle dispersions (~1.5 mg·mL^1^ in cyclohexane) were dropped on copper grids (coated with carbon, 400 mesh) from Plano GmbH. All micrographs were evaluated with the software ImageJ.

# Particle Preparation and Characterization

Oleate (OA) coated UCNPs with core-shell architecture NaYF_4_(20%Yb,2%Er)@NaYF_4_ were synthesized in two different sizes according to well established protocols.^1–3^ Statistical analysis of transmission electron microscopy (TEM) images reveal average diameters of the core-shell UCNPs@OA (12 ± 1) nm (Figure S 1 A) from the (8 ± 2) nm core particles and (33 ± 1) nm (Figure S 1 B) from the (26.3 ± 0.7) nm core particles. The diameters from the TEM analysis were used to determine the number of particles in the suspension to enable comparisons to other particles (Table S 1). The transfer of the UCNPs from cyclohexane to aqueous media was achieved with amphiphilic bilayer surface coatings, using the already attached OA and either an amphiphilic polymer (AP) for UCNPs@AP or a lipid mixture to form a phospholipid membrane (PLM) around the particles UCNPs@PLM. The colloidal stability of the UCNPs@AP and UCNPs@PLM in H_2_O and in the desired media for cell experiments was verified by intensity-weighted dynamic light scattering (DLS) measurements with a narrow distribution (Figure S 1 C,D, Table S 2) and their highly negative zeta potentials in H_2_O (10 mM NaCl) (Table S 2). Luminescence spectra were recorded of all particles in H_2_O upon 980 nm irradiation (cw, 85 W·cm^-1^) (Figure S 1 E). The 12 nm UCNPs have a more pronounced green luminescence with a ratio of green and red luminescence I_g/r_ ~2. Despite the 2 nm thick shell NaYF_4_, the 12 nm UCNPs are still sensitive towards water quenching and the protecting ability and tightness of the particle coatings are visible in the luminescence spectra. The 12 nm UCNPs@PLM are twice as bright as the UCNPs@AP, indicating a better shielding of UCNPs from water by the phospholipid membrane. In contrast, the 33 nm UCNPs with 3 nm thick shell NaYF_4_ have an inverse ratio of green and red fluorescence with I_g/r_ ~0.6, indicating that water quenching, especially of the green emission, is reduced. Moreover, 33 nm UCNPs@AP are almost as bright as the UCNPs@PLM (~‑20%). The homogeneity in luminescence intensities on a single particle level was proven for the 33 nm UCNPs by wide-field upconversion microscopy. Indeed, the intensity distribution of the luminescence spots was homogeneous indicating that most luminescent spots had a similar brightness that could most likely be attributed to the signal of individual UCNPs. The fact that most luminescence spots had similar intensity is only an indirect proof that they correspond to single UCNPs. A direct proof requires to perform time consuming correlative size and luminescence measurement, such as AFM and luminescence measurements that were performed in our previous work that allowed us to calibrate our microscope.^4^ A gaussian fit of the luminescence intensity distribution provided the mean luminescence of individual x_c_(UCNPs@AP) = 2,530 ± 30 cts (N = 894) and individual x_c_(UCNPs@PLM) = 3,220 ± 400 cts  (N = 1,509) (Figure S 1 F). On average, a single 33 nm UCNPs@AP is almost as bright as a 33 nm UCNPs@PLM, matching the ensemble results in solution. The intensity of single 12 nm UCNPs@AP is too low for robust measurements of their luminescence at the single particle scale, making them so far unsuitable for cellular microscopy studies. As homogeneity in luminescence intensity and size distribution could be verified in detail for 33 nm UCPNs by DLS and wide-field upconversion microscopy, these particles were mostly used for comparison studies.

# Supplementary Figures and Tables

**Table S1.** Overview of mass concentration (β), number of particles per mL (N_UCNPs_/ mL) and molar concentration of the 12 nm and 33 nm UCNPs.

| **β(UCNPs) / µg·mL^-1^** | | **d_TEM_ / nm** | **N_UCNPs_ / mL** | **c(UCNPs) / nM** |
| --- | --- | --- | --- | --- |
| 0.1 | 12 | | 2·10^13^ | 35 |
| 0.1 | 33 | | 1·10^12^ | 1.7 |

**Table S2.** Hydrodynamic diameters d_hydro_ and zeta potential ζ of 12 nm as well as 33 nm UCNPs@AP and UCNPs@PLM in H_2_O and in the media for cell experiments: L-15 (5% FCS), DPBS (5% FCS, 1 mg∙mL^−1^ glucose), or cell culture medium DMEM (5% FCS).

|  | **UCNPS@AP** | **UCNPs@PLM** | **UCNPs@AP** | **UCNPs@PLM** |
| --- | --- | --- | --- | --- |
|  | **12 nm** | | **33 nm** | |
| **d_hydro_(H_2_O)/nm** | 22.60 ± 0.08  (PdI = 0.09 ± 0.01) | 60.8 ± 0.1  (PdI = 0.16 ± 0.01) | 56.0 ± 0.6  (PdI = 0.12 ± 0.02) | 125 ± 2  (PdI = 0.19 ± 0.01) |
| **ζ / mV** | -29 ± 2 | -41 ± 3 | -19.3 ± 0.3 | -63 ± 3 |
| **d_hydro_(L-15)/nm** | 50.1 ± 0.5  (PdI = 0.27 ± 0.01) | 140 ± 1  (PdI = 0.17 ± 0.01) | 97 ± 7  (PdI = 0.30 ± 0.01) |  |
| **d_hydro_(DPBS)/nm** |  |  | 93 ± 2  (PdI = 0.26 ± 0.01) | 148 ± 4  (PdI = 0.19 ± 0.02) |
| **d_hydro_(DMEM)/nm** |  | 81 ± 2  (PdI = 0.27 ± 0.01) |  | 180 ± 6  (PdI = 0.12 ± 0.02) |


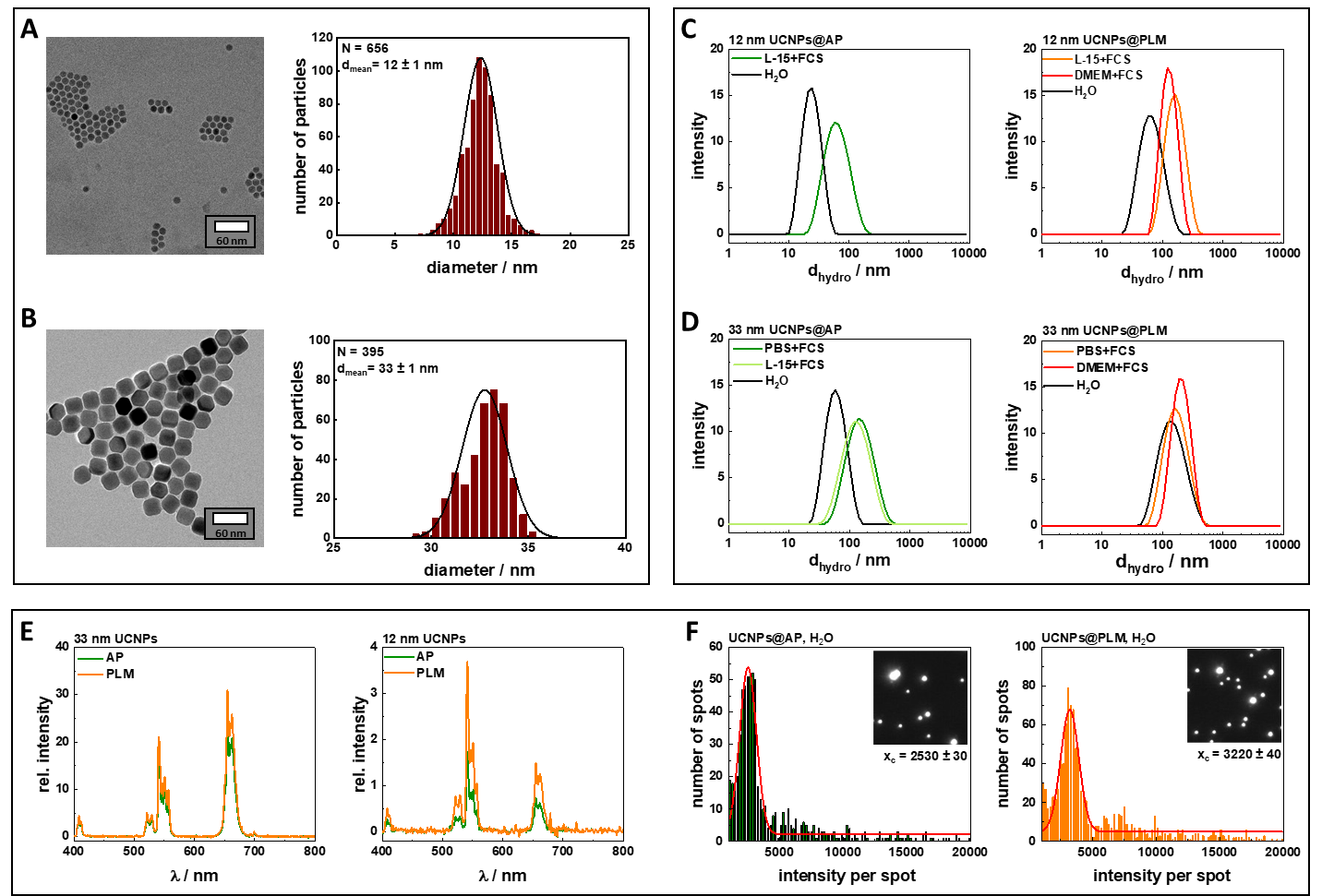


**Figure S1.** Characterization of 12 nm and 33 nm core-shell UCNPs NaYF_4_(20%Yb,2%Er)@NaYF_4_ with size evaluation of unmodified UCNPs@OA (box left), hydrodynamic diameters of UCNPs@AP and UCNPs@PLM (box right) and photophysical properties of UCNPs@AP and UCNPs@PLM (box below). Box left: Transmission electron micrographs (scale bar 60 nm) and corresponding particle size distribution of (A) 12 ± 1 nm and (B) 33 ± 1 nm of OA core-shell UCNPs NaYF_4_(Yb,Er)@NaYF_4_in cyclohexane. Box right: Intensity weighted particle size distribution of the (C) 12 nm and (D) 33 nm UCNPs@AP and UCNPs@PLM in water, DPBS (DPBS + 5% FCS, 1 mg·mL^‑1^ glucose) or Leibovitz L-15 (5% FCS) measured by dynamic light scattering (400 µg·mL^‑1^). Box below: E) Luminescence spectra of 12 nm and 33 nm UCNPs@AP and UCNPs@PLM in H_2_O (400 µg·mL^‑1^) upon 980 nm irradiation (cw, 85 W·cm^‑1^, normalized to N_UCNPs_). F) Luminescence intensity distribution of individual 33 nm UCNPs@AP and UCNPs@PLM in an aqueous solution of 1 mM NaF with representative wide-field upconversion micrographs (λ_exc_ = 974 nm, 8 kW∙cm^‑2^, cw).


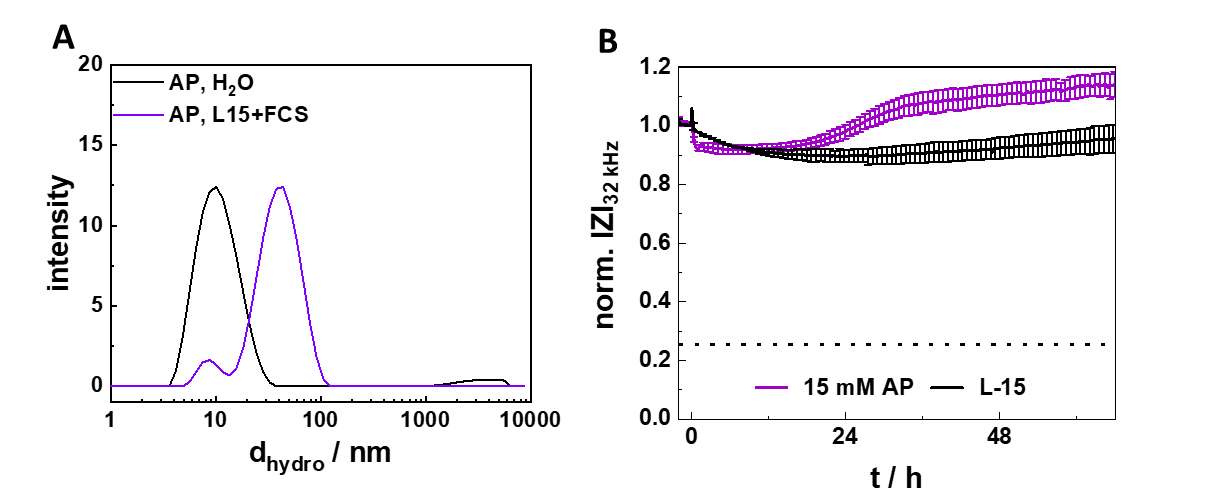


**Figure S2**. (A) Intensity weighted DLS of AP aggregates without UCNPs (15 mM) in H_2_O (d_hydro_= 9.9 ± 0.1 nm, PdI = 0.29 ± 0.01) or in L-15 (5% FCS) (d_hydro_= 30.9 ± 0.9 nm, PdI = 0.24 ± 0.01). (B) Averaged and normalized impedance time courses of NRK cells (recorded at 32 kHz), incubated with AP w/o UCNPs (15 mM monomer concentration, L­‑15, 5% FCS) at time point 0 h. Dashed lines represent cell-free impedance level. Average baseline impedance magnitude was (1.084 ± 0.007) kΩ before addition.


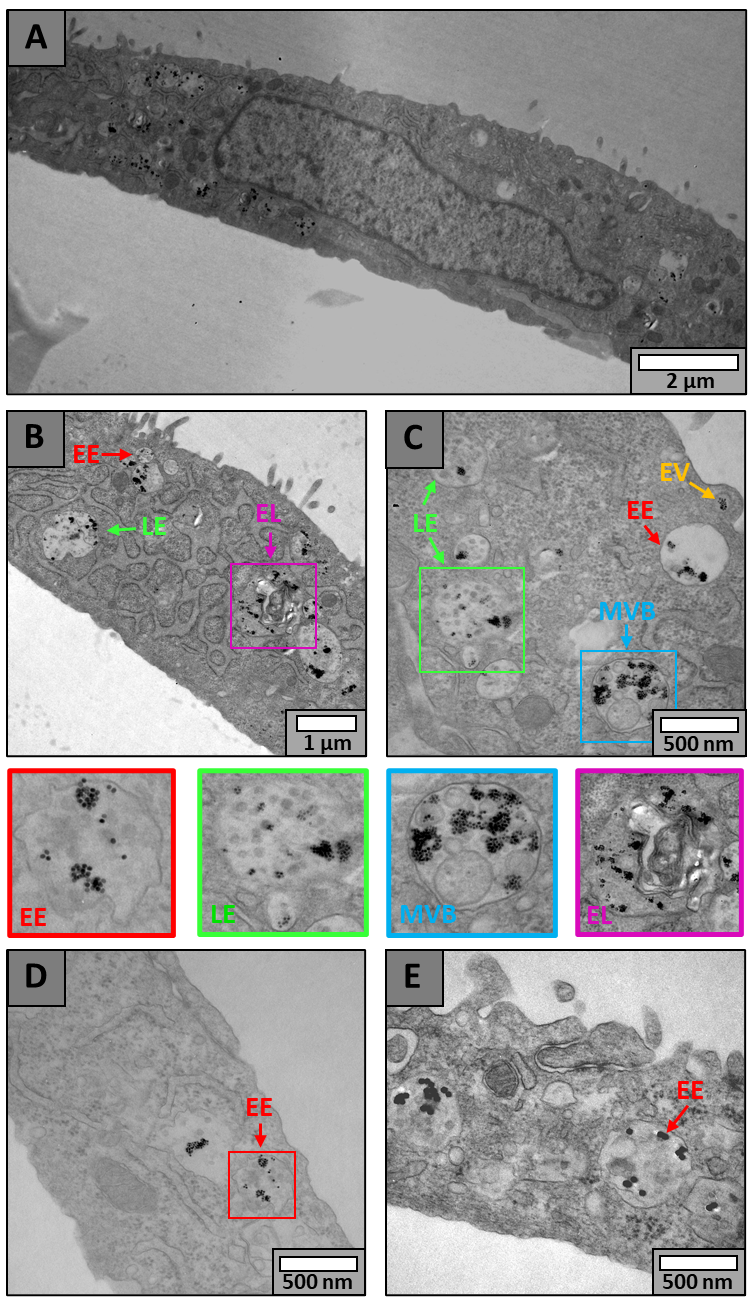


**Figure S3.** Representative electron micrographs captured for 70 nm ultrathin sections of NRK cells incubated with 12 nm UCNPs@PLM (DMEM, 5% FCS) for 24 h (A, B, C) with enlarged insets below. The labels are defined as follows: endocytic vesicles (EV), early endosomes (EE), late endosomes (LE), multivesicular bodies (MVB) and endo-lysosome (EL).

References

(1) Muhr, V.; Würth, C.; Kraft, M.; Buchner, M.; Baeumner, A. J.; Resch-Genger, U.; Hirsch, T. Particle-Size-Dependent Förster Resonance Energy Transfer from Upconversion Nanoparticles to Organic Dyes. *Anal. Chem.* **2017**, *89* (9), 4868–4874. DOI: 10.1021/acs.analchem.6b04662. Published Online: Apr. 18, 2017.

(2) Wilhelm, S.; Kaiser, M.; Würth, C.; Heiland, J.; Carrillo-Carrion, C.; Muhr, V.; Wolfbeis, O. S.; Parak, W. J.; Resch-Genger, U.; Hirsch, T. Water dispersible upconverting nanoparticles: effects of surface modification on their luminescence and colloidal stability. *Nanoscale* **2015**, *7* (4), 1403–1410. DOI: 10.1039/C4NR05954A.

(3) Märkl, S.; Schroter, A.; Hirsch, T. Small and Bright Water-Protected Upconversion Nanoparticles with Long-Time Stability in Complex, Aqueous Media by Phospholipid Membrane Coating. *Nano letters* **2020**, *20* (12), 8620–8625. DOI: 10.1021/acs.nanolett.0c03327. Published Online: Nov. 9, 2020.

(4) Dukhno, O.; Przybilla, F.; Muhr, V.; Buchner, M.; Hirsch, T.; Mély, Y. Time-dependent luminescence loss for individual upconversion nanoparticles upon dilution in aqueous solution. *Nanoscale* **2018**, *10* (34), 15904–15910. DOI: 10.1039/C8NR03892A.
